# Correlates of Adverse Outcomes in Abdominally Obese Individuals: Findings from the Five-Year Followup of the Population-Based Study of Health in Pomerania

**DOI:** 10.1155/2013/762012

**Published:** 2013-09-26

**Authors:** Nele Friedrich, Harald J. Schneider, Ulrich John, Marcus Dörr, Sebastian E. Baumeister, Georg Homuth, Uwe Völker, Henri Wallaschofski

**Affiliations:** ^1^Institute of Clinical Chemistry and Laboratory Medicine, University Medicine Greifswald, Ferdinand-Sauerbruch-Straße NK, 17475 Greifswald, Germany; ^2^German Centre for Cardiovascular Research (DZHK), Partner Site Greifswald, 13347 Berlin, Germany; ^3^Medizinische Klinik—Innenstadt, Ludwig-Maximilians University, Ziemssenstraße 1, 80336 Munich, Germany; ^4^Institute of Epidemiology and Social Medicine, University Medicine Greifswald, Walther-Rathenau-Straße 48, 17475 Greifswald, Germany; ^5^Department of Cardiology, University Medicine Greifswald, Ferdinand-Sauerbruch-Straße NK, 17475 Greifswald, Germany; ^6^Institute for Community Medicine, University Medicine Greifswald, Walther-Rathenau-Strße 48, 17475 Greifswald, Germany; ^7^Interfaculty Institute for Genetics and Functional Genomics, University Medicine Greifswald, Friedrich-Ludwig-Jahn-Straße 15a, 17475 Greifswald, Germany

## Abstract

*Background*. Abdominal obesity is a major risk factor of cardiovascular disease (CVD), type 2 diabetes (T2DM), and premature death. However, it has not been resolved which factors predispose for the development of these adverse obesity-related outcomes in otherwise healthy individuals with abdominal obesity. *Methods*. We studied 1,506 abdominal obese individuals (waist-to-height ratio (WHtR) ≥ 0.5) free of CVD or T2DM from the population-based Study of Health in Pomerania and assessed the incidence of CVD or T2DM after a five-year followup. Logistic regression models were adjusted for major cardiovascular risk factors and liver, kidney diseases, and sociodemographic status. *Results*. During follow-up time, we observed 114 and 136 new T2DM and CVD cases, respectively. Regression models identified age, waist circumference, serum glucose, and liver disease as predictors of T2DM. Regarding CVD, only age, unemployment, and a divorced or widowed marital status were significantly associated with incident CVD. In this subgroup of obese individuals blood pressure, serum glucose, or lipids did not influence incidence of T2DM or CVD. *Conclusion*. We identified various factors associated with an increased risk of incident T2DM and CVD among abdominally obese individuals. These findings may improve the detection of high-risk individuals and help to advance prevention strategies in abdominal obesity.

## 1. Introduction

Obesity is a major risk factor for cardiovascular diseases (CVD), type 2 diabetes mellitus (T2DM), and all-cause mortality [1, 2]. Identification of obese individuals with an unfavourable cardiovascular and metabolic risk profile is important when aiming to identify those who will benefit most from lifestyle interventions or medical treatment. The concept of “metabolically healthy obesity” (MHO), defined as the absence of cardiometabolic risk factors and/or insulin resistance in subjects with a body mass index (BMI) of 30 or greater, was introduced to address different risk profiles [3, 4]. A study of the National Health and Nutrition Examination Survey (NHANES) III revealed a higher mortality risk in unhealthy obese but not in MHO subjects compared to lean subjects [5]. However, this concept does not distinguish between overall and abdominal obesity. 

Recent studies have shown that measures of abdominal obesity, compared with BMI, are better predictors of cardiovascular risk and mortality [6, 7]. The waist-to-height ratio (WHtR) represents a marker with high predictive performance and does not underestimate the amount of body fat in short subjects and overestimates it in tall subjects [8]. Moreover, comparing metabolically healthy and unhealthy obese individuals, the latter showed lower waist circumferences but no differences in BMI [9]. This finding suggests that the presence of cardiometabolic risk factors in obesity may be driven by abdominal fat accumulation [9]. Therefore, it is of interest to reveal factors that are associated with metabolically healthy obesity if alternative measures are used instead of BMI.

Therefore, the present study aims to assess potential predictors of incident T2DM or CVD and cardiovascular death in individuals with abdominal obesity defined by elevated WHtR using data from the longitudinal population-based Study of Health in Pomerania (SHIP).

## 2. Materials and Methods

### 2.1. Subjects

SHIP is a representative, population-based cohort study in West Pomerania, a region in northeast Germany [10, 11]. The total population of West Pomerania selected for SHIP comprised 212,157 inhabitants. A two-stage cluster sampling method adopted from the WHO MONICA Project Augsburg, Germany, yielded 12 five-year-age strata (20 to 79 years) for both sexes, each including 292 individuals. The sampling was performed from population registries, where all German citizens are followed. Data collection started in October 1997 and was finished in March 2001. The net sample (after exclusion of migrated or deceased persons) comprised 6,267 eligible subjects. All subjects received a maximum of three written invitations. In cases of nonresponse, letters were followed by phone calls or home visits if contact by phone was not possible. Finally, 4,308 subjects participated in a health examination in one of two centres that had been established for this purpose (response proportion 69%). Baseline data collection started in October 1997 and was finished in March 2001 [11]. All participants were invited to a follow-up health examination five years later. Vital status information was acquired by death certificates at annual intervals from the time of enrollment until five years of follow up. All participants had given written informed consent. The study conformed to the principles of the Declaration of Helsinki and had been approved by the Ethics Committee of the University of Greifswald. 

At five-year followup, a total of 3,300 individuals participated and further 52 died due to CVD within the first five years. Of these 3,352 subjects, 1967 had been abdominal obese at baseline (WHtR ≥ 0.5) and had complete datasets. Individuals with prevalent T2DM or CVD (angina pectoris, peripheral artery disease, stroke, or myocardial infarction) at baseline (*n* = 461) were excluded. Thus, the final study population consisted of 1,506 individuals (628 women). For comparison, data of 1,128 nonabdominal obese subjects with no prevalent T2DM or CVD at baseline were used.

### 2.2. Clinical Assessments

Information on age, sex, sociodemographic characteristics, and medical history was collected with computer-assisted personal interviews. Smoking status was assessed by self-report, and subjects were categorized as either current smokers or nonsmokers. Subjects who participated in physical activity for at least two hours per week were classified as being physically active. Average alcohol consumption (in grams per day) was calculated by multiplying frequency and amount of pure alcohol from beer, wine, and spirits, respectively, using a standard ethanol content of 4.8% (by volume) in beer, 11% (by volume) in wine, and 33% (by volume) in spirits to conversion [12].

Anthropometric characteristics were measured according to written standardized instructions in accordance with the World Health Organization standards (WHO 1987). Waist circumference was measured to the nearest 0.1 cm midway between the lower rib margin and the iliac crest in the horizontal plane, using an inelastic tape measure. The following anthropometric parameters were calculated: BMI (weight in kg divided by the square of height in meters) and WHtR (waist circumference divided by measured height in cm). Blood pressure was measured three times with an appropriate-sized cuff after five minutes of rest in a sitting position. The mean of the second and third measurement was recorded. Hypertension was defined as systolic or diastolic blood pressure of ≥140 mmHg or ≥90 mmHg, respectively, or intake of antihypertensive medication. Liver disease was defined as self-reported physician's diagnosis of liver disease or aspartate amino transferase (ASAT) or alanin-amino-transferase (ALAT) > population mean + 2 ∗ standard deviation. The definition of T2DM was based on self-reported physician's diagnosis or self-reported use of antidiabetic medication (anatomic, therapeutic, and chemical (ATC) code: A10) in the last 7 days, or glycated hemoglobin (HbA1c) >6.5%. CVD definition was based on a self-reported history of angina pectoris, peripheral artery disease, stroke, or myocardial infarction. Information on vital status was acquired at annual intervals from the time of enrolment through March 8, 2008. Death certificates were requested from the local health authority of the residence of death, and were coded by a certified nosologist according to the International Classification of Diseases, 10th revision (ICD10). CVD death included codes I10 to I79.

### 2.3. Laboratory Measurements

Nonfasting blood samples were drawn from the cubital vein, with the patient in the supine position. The samples were taken between 07:00 a.m. and 06:00 p.m. and analyzed immediately or stored at −80°C until measurement. Total and high-density lipoprotein (HDL) cholesterol concentrations were measured photometrically (Hitachi 704, Roche, Mannheim, Germany). Serum low-density lipoprotein (LDL) cholesterol was measured by applying a precipitation procedure using dextran sulphate (Immuno, Heidelberg, Germany) on an Epos 5060 (Eppendorf, Hamburg, Germany). Glucose concentrations were determined enzymatically using reagents from Roche Diagnostics (Hitachi 717, Roche Diagnostics, Mannheim, Germany). HbA1c concentrations were determined by high-performance liquid chromatography (Bio-Rad Diamat, Munich, Germany). All assays were performed according to the manufacturers' recommendations by skilled technical personnel. In addition, the laboratory participated in official quarterly German external proficiency testing programs.

### 2.4. Statistical Analyses

Abdominal obesity was defined by a WHtR ≥0.5 [13, 14]. We assessed the five-year incidence of CVD (incident CVD events and death due to CVD within the first five years) and T2DM. We performed logistic regression analyses modelling the following predictors of the single endpoints as well as a combined endpoint in individuals with abdominal obesity: smoking status, physical activity, alcohol consumption, hypertension, total cholesterol, LDL cholesterol, HDL cholesterol, liver disease, marital status (married, single, divorced or widowed), and employment status (employed or retired versus unemployed). All models were calculated after adjustment for age and waist circumference. In a further step, we included all predictors in a multivariate model using blockwise selection (I: socio-demographic parameters; II: lifestyle parameters, and III health parameter with *P* value threshold <0.2), with age, sex, and waist circumference forced into the model. A two-sided *P* value of <0.05 was considered statistically significant. All statistical analyses were performed using SAS 9.1 (SAS Institute Inc., Cary, NC, USA). Since the population was selected using a complex sample procedure, the procedures proc surveylogistic and proc surveyfreq were performed using sample weights as well as cluster and strata information to get proper estimates and confidence intervals of estimates.

## 3. Results

In our overall study population of 2,634 individuals free of diabetes and CVD at baseline, 57.2% (*N* = 1,506) were abdominally obese. The baseline characteristics of abdominally obese and nonobese individuals are presented in Table 1. Abdominally obese subjects were older and more often male, smoked less often, and were more often affected by liver disease and hypertension than abdominally nonobese participants. The incidence of the CVD or T2DM was higher in abdominal obese subjects compared to nonobese subjects (CVD: obese; 9.0% (*N* = 136), age-adjusted: 8.0%; nonobese 3.4% (*N* = 38), age-adjusted: 4.7%; T2DM: obese; 7.7% (*N* = 114), age-adjusted: 6.5%; nonobese 0.7% (*N* = 8), age-adjusted: 2.2%). After adjustment for age, sex, and waist circumference, positive associations were found between a divorced or widowed marital status, unemployment, hypertension, total cholesterol, LDL cholesterol, glucose levels or blood pressure and the incidence of CVD (Table 2). With respect to T2DM, liver disease and glucose serum concentration were positively and HDL cholesterol levels negatively associated with incidence of T2DM (Table 2). Further, in multivariate logistic regression models the blockwise selection procedure yielded only a male gender, high age, unemployment and a divorced or widowed marital status as significant predictors (*P* < 0.05) of incident CVD (Table 3). No health related parameters like hypertension or systolic blood pressure were significant CVD predictors in abdominal obese subjects. Regarding incident T2DM the following significant predictors (*P* < 0.05) were revealed: a high age and waist circumference as well as high glucose or HDL cholesterol levels, and liver disease (Table 3). These predictors except HDL cholesterol were also found for the combined endpoint, even if liver disease barely missed statistical significance (each *P* = 0.06). Furthermore, unemployment was related to a higher risk of T2DM or CVD.

**Figure 1:**
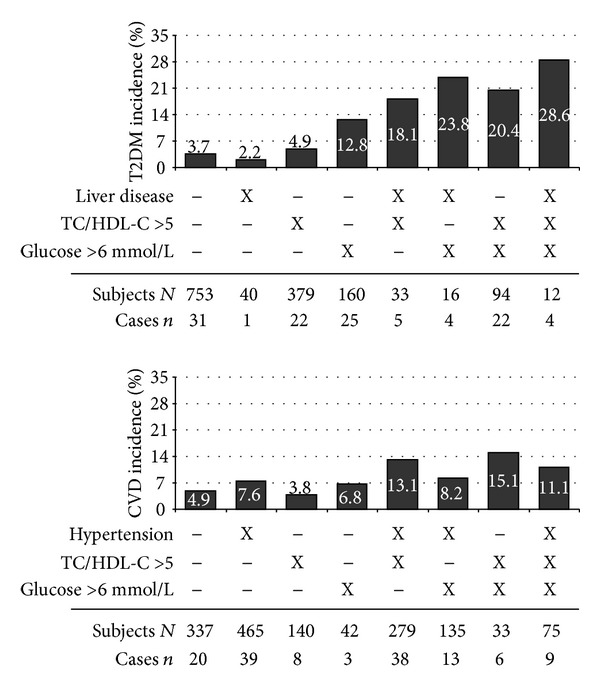
Incidence of type 2 diabetes mellitus (T2DM) and cardiovascular disease (CVD) depending on important risk factors including liver disease (only for diabetes), hypertension (only for CVD), increased total cholesterol—high-density lipoprotein cholesterol ratio (TC/HDL-C > 5), and increased glucose levels (>6 mmol/L).

To illustrate the importance of health parameters among abdominal obese subjects the incidences of T2DM or CVD are displayed depending on biomarkers like increased glucose levels (>6 mmol/L), increased total cholesterol—HDL cholesterol ratio (>5), and liver disease (diabetes) or hypertension (CVD) (Figure 1).

## 4. Discussion

We found that 15.6% of disease-free individuals with abdominal obesity develop T2DM or CVD within the first five years of follow-up. The present study identified high age, high waist circumference, liver disease and high glucose levels as significant predictors of incident T2DM. With respect to incident CVD, high age, unemployment, and the marital status were identified as significant predictors. Interestingly, parameter like blood pressure or glucose and lipid levels did not constitute CVD predictors in abdominal obese subjects. This is the first large study describing healthy obesity in a population free of obesity-related comorbidities including T2DM and CVD at baseline and applying WHtR instead of BMI as measure of abdominal obesity.

BMI displays a J-shaped association with death and cardiovascular risk, whereas measures of abdominal obesity show a linear association with these risks after adjustment for BMI [7, 15]. Moreover, measures of abdominal obesity have been shown to outperform the predictive utility of BMI with regard to cardiovascular risk and mortality [6, 7]. Even though waist circumference is the most commonly used measure of abdominal obesity, we used the WHtR to define abdominally obese and lean states for two reasons: (1) assessing cardiovascular risk, the comparative predictive performance of WHtR was higher than other measures of abdominal obesity not only in the present study but also in other prospective observational studies [6, 7]. (2) If waist circumference is used with a defined cutoff (e.g., 102 cm in men), the waist circumference underestimates the amount of body fat in short subjects and overestimates it in tall subjects [8]. This is not the case for the WHtR. Some of the factors we found to be associated with the development of the combined endpoint represent well-known cardiovascular risk factors [16] including higher age, glucose, and total cholesterol levels. BMI was an additional independent predictor, indicating that overall obesity apart from abdominal obesity plays an additional role in the development of obesity-related events.

Furthermore, we found that liver disease predicted T2DM but not CVD. Nonalcoholic fatty liver disease is the main cause of elevated liver enzymes and is associated with T2DM [17] and has been shown to be an independent risk factor of CVD [18, 19]. Unemployment predicted CVD independently of other risk factors. A potential explanation for this particular finding in our study might be the high unemployment rate in the study region compared to other regions in Germany. In the PRIME studies [20], unemployment predicted cardiovascular risk after adjustment for age but not after adjustment for other cardiovascular risk factors.

Several strengths and limitations of our study need to be addressed. Our approach allowed us to identify factors directly associated with obesity-related outcomes in abdominally obese individuals over a defined time span. Limitations include that our approach does not allow conclusions on a timespan beyond 5 years. We chose a follow-up period of five years because we think this is a time span relevant to the clinician. Moreover, we do not know if our results are generalizable to other populations or risk groups. A further limitation arises from the fact that the assessment of prevalent and incident CVD and T2DM based on self-reported diagnosis, which might lead to a reduced validity due to misclassification. However, several studies revealed a good agreement between self-reported diseases and medical record or clinical definitions [21–24]. The subjects in our study were not fasting, which should be considered particularly with respect to glucose levels. Therefore, a direct measure of insulin sensitivity was not possible in this cohort. However, we included Hba1c, a measure of long-term glucose load. The parameters we used were easy assessable and allowed a direct assessment of obesity-related risk. 

## 5. Conclusion

In the present study, we identified a set of factors including age, waist circumference, liver disease, glucose levels and unemployment as predictors of obesity-related diseases in abdominal obese subjects. These predictors might allow stratification of subjects and adaptation prevention and therapeutic strategies in the future.

## Figures and Tables

**Table 1:** Baseline characteristics of the study population.

Characteristics	WHtR < 0.5 (*N* = 1,128)	WHtR ≥ 0.5 (*N* = 1,506)	P*
Age, years	37 (29; 48)	54 (42; 63)	<0.01
Men, %	31.8	58.3	<0.01
Current smokers, %	34.0	26.8	<0.01
Physical active, %	54.4	40.6	<0.01
Employment, %			<0.01
Employed	72.0	50.3	
Retired	10.7	35.7	
Unemployed	17.3	14.0	
Marital status, %			<0.01
Married	57.1	74.3	
Divorced or widowed	12.4	14.0	
Single	30.5	11.7	
School education, %			<0.01
<10 years	16.2	42.2	
10 years	60.0	42.5	
>10 years	23.8	15.3	
Liver disease. %	2.3	6.9	<0.01
Body mass index, kg/m^2^	23.1 (21.6; 24.8)	28.8 (26.9; 31.5)	<0.01
Waist circumference, cm	76 (70; 81)	95 (90; 102)	<0.01
Alcohol consumption, g/day	5.0 (0; 13.6)	5.4 (0; 19.8)	0.03
Total cholesterol, mmol/L	5.3 (4.6; 6.0)	6.0 (5.3; 6.7)	<0.01
HDL cholesterol, mmol/L	1.6 (1.3; 1.9)	1.3 (1.1; 1.6)	<0.01
LDL cholesterol, mmol/L	3.0 (2.4; 3.8)	3.8 (3.1; 4.5)	<0.01
Glucose, mmol/L	5.0 (4.7; 5.4)	5.4 (5.0; 5.8)	<0.01
Systolic blood pressure, mm Hg	123 (113; 134)	139 (128; 152)	<0.01
Diastolic blood pressure, mm Hg	78 (72; 84)	87 (80; 94)	<0.01
Hypertension, %	22.4	63.4	<0.01
Incident CVD, %	3.4	9.0	<0.01
Incident T2DM, %	0.7	7.7	<0.01

CVD: cardiovascular disease; T2DM: type 2 diabetes mellitus; HDL: high-density lipoprotein; LDL: low-density lipoprotein. Continuous data are expressed as median (25th; 75th percentile); nominal data are given as percentages. *χ ^2^-test (nominal data) and Mann-Whitney test (interval data) were performed.

**Table 2:** Association between potential predictors and cardiovascular disease (CVD) or type 2 diabetes mellitus (T2DM) among abdominal obese individuals calculated by logistic regression models adjusted for sex, age, and waist circumference.

	Incident CVD		Incident T2DM		Incident T2DM or CVD	
	OR (95% CI)	P*	OR (95% CI)	P*	OR (95% CI)	P*
*N* (cases)	1506 (136)		1490 (114)		1490 (234)	
*Sociodemographic parameters *						
Unemployment	2.43 (1.39; 4.25)	<0.01	1.03 (0.53; 1.97)	0.94	1.83 (1.12; 2.97)	0.02
Marital status (ref: married)						
Divorced or widowed	1.89 (1.24; 2.88)	<0.01	1.32 (0.58; 3.00)	0.50	1.69 (1.02; 2.80)	0.04
Single	1.47 (0.82; 2.65)	0.20	1.46 (0.49; 4.39)	0.50	1.65 (0.85; 3.19)	0.14
*Lifestyle parameters *						
Current smoking	1.10 (0.72; 1.68)	0.67	1.09 (0.76; 1.56)	0.64	1.04 (0.75; 1.42)	0.83
No physical activity	0.94 (0.45; 1.96)	0.88	1.31 (0.74; 2.33)	0.36	1.11 (0.61; 2.02)	0.72
*Health parameters *						
Liver disease	1.31 (0.68; 2.49)	0.42	2.67 (1.18; 6.04)	0.02	1.97 (1.09; 3.57)	0.02
Hypertension	1.43 (1.05; 1.95)	0.02	1.18 (0.76; 1.83)	0.47	1.39 (1.16; 1.67)	<0.01
Alcohol consumption (ref: Q1)		0.93		0.21		0.44
Q2	1.04 (0.59; 1.83)		0.81 (0.38; 1.71)		0.91 (0.49; 1.69)	
Q3	1.02 (0.67; 1.56)		0.99 (0.63; 1.54)		0.94 (0.63; 1.39)	
Q4	1.02 (0.67; 1.56)		0.64 (0.38; 1.06)		0.85 (0.61; 1.19)	
Total cholesterol (ref: Q1)		0.01		0.13		0.12
Q2	1.11 (0.74; 1.66)		0.49 (0.24; 1.02)		0.84 (0.54; 1.29)	
Q3	1.22 (0.73; 2.03)		0.54 (0.29; 0.98)		0.88 (0.62; 1.26)	
Q4	1.89 (1.20; 2.99)		0.67 (0.43; 1.05)		1.30 (0.94; 1.79)	
Total cholesterol, per SD increase	1.39 (1.18; 1.65)	<0.01	—		1.18 (1.04; 1.36)	0.01
HDL cholesterol (ref: Q4)		0.64		<0.01		0.03
Q1	1.11 (0.71; 1.73)		2.39 (1.43; 3.99)		1.46 (1.04; 2.05)	
Q2	1.07 (0.65; 1.78)		1.46 (0.84; 2.53)		1.29 (0.94; 1.77)	
Q3	1.05 (0.65; 1.68)		1.58 (0.88; 2.84)		1.24 (0.97; 1.60)	
HDL cholesterol, per SD decrease	—		1.32 (1.11; 1.56)	<0.01	1.12 (1.01; 1.25)	0.04
LDL cholesterol (ref: Q1)		0.04		0.22		0.13
Q2	1.03 (0.65; 1.61)		0.53 (0.25; 1.08)		0.85 (0.54; 1.35)	
Q3	0.99 (0.58; 1.69)		0.55 (0.25; 1.22)		0.83 (0.54; 1.26)	
Q4	1.62 (1.06; 2.47)		0.74 (0.48; 1.14)		1.24 (0.95; 1.62)	
LDL cholesterol, per SD increase	1.37 (1.15; 1.64)	<0.01	—		1.19 (1.06; 1.34)	<0.01
Glucose (ref: Q1)		0.05		<0.01		<0.01
Q2	1.43 (0.90; 2.28)		1.72 (1.01; 2.94)		1.66 (1.12; 2.45)	
Q3	2.01 (1.37; 2.95)		1.51 (0.74; 3.09)		2.02 (1.48; 2.77)	
Q4	1.50 (0.88; 2.54)		4.29 (2.45; 7.51)		2.77 (1.84; 4.16)	
Glucose, per SD increase	1.14 (1.05; 1.23)	<0.01	1.89 (1.43; 2.49)	<0.01	1.56 (1.35; 1.79)	<0.01
Systolic BP (ref: Q1)		0.03		0.71		0.07
Q2	1.52 (1.10; 2.09)		1.22 (0.71; 2.11)		1.35 (1.00; 1.84)	
Q3	1.67 (1.17; 2.39)		0.79 (0.41; 1.53)		1.23 (0.88; 1.71)	
Q4	2.05 (1.16; 3.63)		1.01 (0.62; 1.65)		1.55 (1.09; 2.20)	
Systolic BP, per SD increase	1.23 (1.00; 1.51)	0.05			1.17 (1.02; 1.34)	0.02
Diastolic BP (ref: Q1)		0.02		0.79		0.10
Q2	0.98 (0.59; 1.62)		0.56 (0.25; 1.26)		0.70 (0.37; 1.32)	
Q3	1.24 (0.71; 2.15)		0.74 (0.35; 1.60)		1.02 (0.54; 1.94)	
Q4	1.50 (0.97; 2.31)		0.96 (0.60; 1.53)		1.22 (0.81; 1.83)	

Abdominal obesity was defined by WHtR ≥ 0.5. OR: odds ratio; CI: confidence interval; HDL: high-density lipoprotein; LDL: low-density lipoprotein; BP: blood pressure; SD: standard deviation.

**Table 3:** Predictors of cardiovascular disease (CVD) or type 2 diabetes mellitus (T2DM) in abdominal obese individuals retained after blockwise selection in multivariate analysis.

	Incident CVD		Incident T2DM		Incident T2DM or CVD	
	OR (95% CI)	*P*	OR (95% CI)	*P*	OR (95% CI)	*P*
*N* (cases)	1506 (136)		1490 (114)		1490 (114)	
Male	1.37 (1.00; 1.87)	0.05	1.28 (0.76; 2.14)	0.35	1.30 (0.97; 1.73)	0.08
Age, per years	1.04 (1.02; 1.05)	<0.01	1.05 (1.03; 1.07)	<0.01	1.04 (1.03; 1.06)	<0.01
Waist circumference, per cm	1.01 (0.99; 1.03)	0.24	1.07 (1.05; 1.09)	<0.01	1.04 (1.03; 1.05)	<0.01
*Sociodemographic parameters *						
Unemployment	2.38 (1.35; 4.18)	<0.01	—		1.82 (1.12; 2.95)	0.02
Marital status (ref: married)						
Divorced or widowed	1.80 (1.14; 2.83)	0.01	—		1.54 (0.90; 2.62)	0.12
Single	1.35 (0.74; 2.48)	0.33	—		1.46 (0.71; 3.01)	0.31
*Health parameters *						
Alcohol consumption, per SD increase	—		0.83 (0.64; 1.09)	0.19	—	
Liver disease	—		2.49 (1.01; 6.18)	0.05	1.77 (0.97; 3.23)	0.06
HDL cholesterol, per SD decrease	—		1.21 (1.02; 1.43)	0.03	—	—
Glucose, per SD increase	—		1.83 (1.39; 2.42)	<0.01	1.54 (1.32; 1.79)	<0.01

Abdominal obesity was defined by WHtR ≥ 0.5. OR: odds ratio; CI: confidence interval; HDL: high-density lipoprotein; SD: standard deviation.
